# Trait − driven analysis of the 2p15p16.1 microdeletion syndrome suggests a complex pattern of interactions between candidate genes

**DOI:** 10.1007/s13258-023-01369-7

**Published:** 2023-02-20

**Authors:** Martina Miceli, Pinella Failla, Lucia Saccuzzo, Ornella Galesi, Silvestra Amata, Corrado Romano, Maria Clara Bonaglia, Marco Fichera

**Affiliations:** 1 Department of Biomedical and Biotechnological Sciences, Medical Genetics, University of Catania, Catania, Italy; 2 Oasi Research Institute−IRCCS, Troina, Italy; 3 Research Unit of Rare Diseases and Neurodevelopmental Disorders, Oasi Research Institute−IRCCS, Troina, Italy; 4 Cytogenetics Laboratory, Scientific Institute, IRCCS Eugenio Medea, Bosisio Parini, Lecco, Italy

**Keywords:** 2p15p16.1 microdeletion syndrome, Epistasis, Additive effect, Penetrance, Neurodevelopmental delay

## Abstract

**Background:**

Individuals with the 2p15p16.1 microdeletion syndrome share a complex phenotype including neurodevelopmental delay, brain malformations, microcephaly, and autistic behavior. The analysis of the shortest region of overlap (SRO) between deletions in ~ 40 patients has led to the identification of two critical regions and four strongly candidate genes (*BCL11A, REL, USP34* and *XPO1*). However, the delineation of their role in the occurrence of specific traits is hampered by their incomplete penetrance.

**Objective:**

To better delineate the role of hemizygosity of specific regions in selected traits by leveraging information both from penetrant and non − penetrant deletions.

**Methods:**

Deletions in patients that do not present a specific trait cannot contribute to delineate the SROs. We recently developed a probabilistic model that, by considering also the non − penetrant deletions, allows a more reliable assignment of peculiar traits to specific genomic segments. We apply this method adding two new patients to the published cases.

**Results:**

Our results delineate an intricate pattern of genotype − phenotype correlation where *BCL11A* emerges as the main gene for autistic behavior while *USP34* and/or *XPO1* haploinsufficiency are mainly associated with microcephaly, hearing loss and IUGR. *BCL11A, USP34* and *XPO1* genes are broadly related with brain malformations albeit with distinct patterns of brain damage.

**Conclusions:**

The observed penetrance of deletions encompassing different SROs and that predicted when considering each single SRO as acting independently, may reflect a more complex model than the additive one. Our approach may improve the genotype/phenotype correlation and may help to identify specific pathogenic mechanisms in contiguous gene syndromes.

## Introduction

The 2p15p16.1 microdeletion syndrome is a rare contiguous gene syndrome characterized by delayed psychomotor development, intellectual disability (ID), and variable but distinctive dysmorphic features, including bitemporal narrowing, smooth and long philtrum, hypertelorism, downslanting palpebral fissures, broad nasal root, thin upper lip, and high palate. Other less penetrant traits are autism spectrum disorder (ASD), structural brain abnormalities, microcephaly, hearing loss, intrauterine growth restriction, and short stature.

Rajcan − Separovic et al. first reported two unrelated patients carrying respectively a 6.1 and 7.9 Mb deletions on chromosome 2 at positions 56,919,993 − 63,032,165 and 55,627,639 − 63,519,476 (hg19). Both patients shared several common phenotypic features, including (moderate to severe) ID, ASD, microcephaly, structural brain anomalies, optic nerve hypoplasia and dysmorphic features (Rajcan − Separovic et al. 2007). Two additional articles (De Leeuw et al. 2008; Chabchoub et al. 2008) described further individuals with deletions in 2p15p16. These reports confirmed the presence of some clinical features common to all patients, as ID and dysmorphic features. Interestingly, Chaochub et al., describing a smaller deletion (0.583 Mb) in a patient lacking some phenotypic traits previously reported by Rajcan − Separovic et al., suggested the possible existence of different candidate genes, each responsible for specific clinical traits.

More recently, several works have been published (Liang et al. 2009; Félix et al. 2010; Piccione et al. 2012; Hucthagowder et al. 2012; Florisson et al. 2013; Hancarova et al. 2013; Fannemel et al. 2014; Jorgez et al. 2014; Balci et al. 2015; Ronzoni et al. 2015; Shimojima et al. 2015; Basak et al. 2015; Ottolini et al. 2015; Bagheri et al. 2016; Lévy et al. 2017; Shimbo et al. 2017), reporting additional patients with highly variable sized deletions ranging from 0.1 to 9.5 Mb, however their distribution does not allow to outline a single Shortest Region of Overlap (SRO) shared from all patients. Indeed, the existence of non − overlapping deletions has made complicated the individuation of a common critical region, responsible for the phenotypic traits of the syndrome.

In fact, deletions reported by Peter et al. and Balci et al. only involved the *BCL11A* gene, suggesting that haploinsufficiency of this gene may be responsible for a subset of traits (such as neurodevelopmental delay, language delay and attention deficit) (Peter et al. 2014; Balci et al. 2015). Interestingly, loss − of − function mutations of this gene have been associated (Dias et al. 2016) with the Dias − Logan syndrome (OMIM: 617,101) characterized by neurodevelopmental delay, facial dysmorphisms and asymptomatic persistence of fetal hemoglobin.

Other articles pinpointed another region of critical importance containing the *USP34* and *XPO1* genes. Indeed, some reported patients (Fannemel et al. 2014; Ronzoni et al. 2015; Shimojima et al. 2015 (patient 1); Bagheri et al. 2016 (patient 6); Lévy et al. 2017 (patient 2)) presented deletions overlapping only these two genes, corroborating the hypothesis of their direct involvement in the disease.

Levy et al. in 2017 classified patients carrying deletions in chromosome 2p15p16.1 in two different groups, according to their exclusive inclusion either of the *BCL11A* gene or the *USP34* and *XPO1* genes. The authors reported that in both groups the most common abnormalities including neurodevelopmental delay, dysmorphic features and brain anomalies were present, although with slightly different frequencies between the two groups (Lévy et al. 2017).

However, incomplete penetrance for several clinical features such as autistic behavior, structural brain anomalies, microcephaly, hearing loss, and IUGR, does not allow to draw a precise genotype/phenotype correlation in different sized deletions, suggesting that other factors, including complex interaction between genes or regulatory elements within deletions, and different individual genetic background, may modulate the final clinical outcome of deletions in that region.

In this work we focus our investigation on selected 2p15p16.1 phenotypes assessed in 36 previously reported patients and in two additional individuals, with the aim of providing more insight into the role of candidate regions in producing a specific phenotype. Since mapping candidate regions for low − penetrant traits is hampered by the fewer number of overlapping penetrant deletions, we used in this study a probabilistic model described in our previous work (Fichera et al. 2020) which tries to gather information also from non − penetrant deletions.

## Materials and methods

### Patients

In addition to our two novel patients, we collected 36 patients carrying 2p15p16.1 deletions from an extensive review of the literature (Rajcan − Separovic et al. 2007; De Leeuw et al. 2008; Chabchoub et al. 2008; Liang et al. 2009; Félix et al. 2010; Piccione et al. 2012; Hucthagowder et al. 2012; Florisson et al. 2013; Fannemel et al. 2014; Jorgez et al. 2014; Peter et al. 2014; Balci et al. 2015; Ronzoni et al. 2015; Shimojima et al. 2015; Basak et al. 2015; Ottolini et al. 2015; Bagheri et al. 2016; Lévy et al. 2017; Shimbo et al. 2017).

Some of the previously reported cases were excluded from our study for several reasons.

The patient described by Prontera et al. was not included because she presented two additional genomic rearrangements, whose contribution to the phenotype was unknown. In particular, the paracentric inversion of chromosome 7 and an apparently balanced translocation between chromosome 1 and 7, involving the same inverted chromosome 7 (Prontera et al. 2011); Bagheri et al. patient 2 was omitted because Array − CGH highlighted a complex rearrangement in the 2p15p16.1 region that would have complicated our analysis. Indeed, he presented two additional smaller CNVs, detected by high resolution array, one mapping in the intronic region of *BCL11A* and another one in the intragenic region proximal to *BCL11A* (Bagheri et al. 2016); Shimojima et al. patient 2, Lévy et al. patient 1, and Jorgez et al. patient 1 were excluded because the inheritance of their deletions was unknown (Jorgez et al. 2014; Shimojima et al. 2015; Lévy et al. 2017).

Jorgez et al. patients 2 and 7 were excluded because their deletions span between the end of the 2p15 and the 2p14 (Jorgez et al. 2014), outside the region of our study.

In all the other patients considered for our study the deletions are reported as de novo*.*

### Estimation of the probability for a given genomic location to overlap the disease locus associated with the selected clinical feature

We used a recently developed bayesian probabilistic model to estimate for each non − overlapping sliding window (Δ) of 1 kb, the posterior probability to intersect the DL, conditioned by the experimental data (i.e., the set of deletions overlapping the specific window inside the SRO).

Briefly, patients were binary classified as showing or not a specific trait and then grouped and analyzed independently for each of the selected traits. Clearly, the number of individuals in each group varied as not all patients were evaluated for each specific trait. In the first step the software identifies the SRO regions, considering overlaps between deletions associated with the trait. By definition, these SROs have probability 1 to contain the DL associated with the selected clinical feature. In the next phase the procedure estimates the probability distribution inside SRO(s) taking into account both penetrant and non − penetrant deletions for the trait.

Finally, for each selected trait the software automatically builds custom bed and bed − graph files to visualize in their genomic context the set of deletions, the probability profiles, and the cumulative probability for each gene inside the SROs. These files were then uploaded and graphically displayed using the genome browser at UCSC (https://genome.ucsc.edu). Accessory files containing the observed penetrance (op) of each genomic windows inside the SROs, and the cumulative probability (cp) for each gene to contain the DL were also created.

In cases of phenotypic traits depicting more than one SRO with incomplete penetrance, we decided to investigate whether penetrances of the DLs inside the SROs might have suggested epistasis or, on the contrary, each single DL was more likely to contribute independently to the trait. At this aim, for deletions encompassing two SROs, we compared the observed penetrances and those expected according to the heterogeneity model (Risch 1990).

$$P_{hm} = P_{SRO1} + P_{SRO2} - P_{SRO1} \cdot P_{SRO2}$$

where Phm represents the probability that an individual with a deletion overlapping both SRO1 and SRO2 is affected through either locus mechanism acting independently.

For our analysis we considered 6 traits (developmental delay, brain abnormalities, IUGR, microcephaly, hearing loss, and autistic behavior).

### Array − CGH investigation

Array − CGH analysis on both patients and their parents was carried out using the SurePrint G3 Custom CGH Microarray, 8 × 60 K 4 × 180 K (Agilent Technologies, Santa Clara, CA, USA) according to the manufacturer’s protocol version 7.1, using appropriate Agilent Reference DNAs (Euro male and Euro female). The arrays were analyzed with the Agilent Microarray Scanner, Feature Extraction Software version 11.5, and Agilent Genomic Workbench 7.0.

## Results

### New patients

Patient 1 (id_723_Troina) is a 25 years old male, born after a normal intrauterine growth. He showed moderate ID, language skills delay and attention deficit. The patient had passive and oppositive behavior. Clinical examination showed hypotonia, scoliosis, coloboma of the retina and dysmorphic features, such as long face and thick lips.

Patient 2 (id_7180_Troina) is a 36 years old female, with normal intrauterine growth and microcephaly. She had moderate ID, global neurodevelopmental delay, language skills delay and attention deficit. The patient showed passive and oppositive behavior. The clinical examination showed hypotonia, scoliosis, pectus excavatum, dental agenesis and dysmorphic features, such as down slanting palpebral fissures, hypertelorism, broad nose, bifid uvula and camptodactyly.

### Genetic characterization

In both patients apparently de novo microdeletions spanning the 2p15p16.1 chromosomic region were found. Patient 1 showed a ~ 140 Kb deletion (arr[GRCh37] 2p15(63058141_63198230) × 1 dn), encompassing the gene *EHBP1*. Patient 2 presented a 1.325 Mb deletion (arr[GRCh37] 2p16.1 p15 (60294104_61618758) × 1 dn) overlapping the 2p16.1 and 2p15 genomic bands and involving the following genes: *MIR4432, BCL11A, MIR562, PAPOLG, FLJ16341, REL, PUS10, PEX13, KIAA1841, LOC339803, AHSA2* and *USP34*.

### Short region of overlaps and probability profiling

Molecular findings and clinical assessment for the 6 selected traits in the 38 patients having deletions (mean 2.70 Mb; median 2.39 Mb) in the 2p15p16.1 region are summarized in Table 1. The rearrangements taken into account extend over a region of 10 Mb (55.61 to 66.37 Mb) including 41 coding genes, ten of which (*EFEMP1, BCL11A, PAPOLG, REL, USP34, XPO1, CCT4, VPS54, AFTPH, ACTR2*) are predicted to be intolerant to loss − of − function mutation (pLI score > 0.95). The region also contains 14 OMIM disease genes, two of which *EFEMP1* (OMIM: 601,548) and *BCL11A* (OMIM: 606,557) are dominant and associated with the Doyne honeycomb degeneration of retina (OMIM: 126,600) and with the Dias − Logan syndrome (OMIM: 617,101), respectively.

**Table 1:** Molecular findings and clinical assessment of patients with 2p15p16.1 microdeletion syndrome

Patient	Start	Stop	Size	Neurodev. Delay	ASD	Microcephaly	Brain anomalies		Hearing loss	IUGR	
Balci_2015_Basak__2015_pat3	59,958,420	60,834,298	875,878	+	na	−	+	−			−
Basak_2015_pat1_Hancarova_2013	60,689,977	61,127,979	438,002	+	+	+	−	−			−
Basak_2015_pat2	60,029,857	61,059,383	1,029,526	+	+	+	−	na			na
Fannemel_2014	61,500,346	61,733,075	232,729	+	na	−	−	+			−
Felix_2010	59,139,200	62,488,871	3,349,671	+	−	+	−	−			+
Ronzoni_2015	61,659,957	61,762,873	102,916	+	na	−	+	na			−
Shimbo_2017_pat1	58,029,768	61,275,725	3,245,957	+	−	−	+	−			−
Shimbo_2017_pat2	60,676,037	65,731,798	5,055,761	+	na	+	+	+			−
Shimbo_2017_pat3	61,136,131	66,258,735	5,122,604	+	−	+	+	+			+
Shimbo_2017_pat4	60,013,464	61,136,190	1,122,726	+	na	−	+	na			−
Shimojima_2015_pat1	61,495,220	61,733,075	237,855	+	na	+	+	−			+
Deleeuw_2007	58,216,217	61,667,426	3,451,209	+	−	+	na	−			−
Piccione_2012_pat1	60,603,496	61,246,496	643,000	+	−	−	−	−			na
Piccione_2012_pat2	60,257,496	62,762,496	2,505,000	+	na	+	+	+			na
Rajcan_Separovic_2007_pat1	56,919,993	63,032,165	6,112,172	+	+	+	+	−			+
Rajcan_Separovic_2007_pat2	55,627,639	63,519,476	7,891,837	+	+	+	+	+			−
Chabchoub_2008	61,203,258	61,786,583	583,325	+	−	−	−	−			−
Florisson_2013_pat1	55,616,146	62,362,249	6,746,103	+	na	+	+	−			na
Florisson_2013_pat2	58,714,795	65,392,528	6,677,733	+	na	+	na	+			na
Peter_2014	60,689,299	60,830,491	141,192	+	−	−	na	−			−
Bagheri_2016_pat1	55,676,099	65,250,541	9,574,442	+	+	+	+	−			+
Bagheri_2016_pat3	59,017,244	64,379,673	5,362,429	+	na	+	−	−			+
Bagheri_2016_pat4	60,650,589	61,621,631	971,042	+	na	−	na	−			na
Bagheri_2016_pat5	61,060,687	65,653,379	4,592,692	+	na	+	na	−			na
Bagheri_2016_pat6	61,438,499	61,797,959	359,460	+	−	+	na	−			+
Bagheri_2016_pat7	61,585,906	64,253,124	2,667,218	+	−	+	na	na			na
Bagheri_2016_pat8	61,739,766	62,534,498	794,732	+	na	−	na	−			−
Levy_2017_pat2	61,671,686	61,777,241	105,555	+	+	−	+	+			−
Levy_2017_pat3	60,624,940	61,051,867	426,927	+	+	−	+	−			−
Liang_2009	59,241,620	62,385,716	3,144,096	+	+	+	−	−			+
Ottolini_2015	60,118,706	61,800,462	1,681,756	+	na	+	na	na			+
Huctagowder_2012	60,672,255	63,144,695	2,472,440	+	na	+	+	+			+
id_723_Troina	63,058,141	63,198,230	140,089	+	−	−	na	−			−
id_7180_Troina	60,294,104	61,618,758	1,324,654	+	−	+	−	−			−
Jorgez_2014_pat4	60,066,496	66,376,960	6,310,464	+	na	−	+	na			na
Jorgez_2014_pat5	61,056,496	65,656,496	4,600,000	+	na	+	na	na			na
Jorgez_2014_pat6	61,566,496	64,316,496	2,750,000	+	na	−	na	na			na
Jorgez_2014_pat3	61,126,496	63,516,496	2,390,000	+	na	+	na	na			na
Penetrances				1.00 (38/38)	0.42 (8/19)	0.61 (23/38)	0.64 (16/25)	0.28 (8/29)			0.38 (10/26)

A*SD* autism spectrum disorder, *IUGR* intrauterine growth restriction, *na* not assessed

**Fig. 1:**
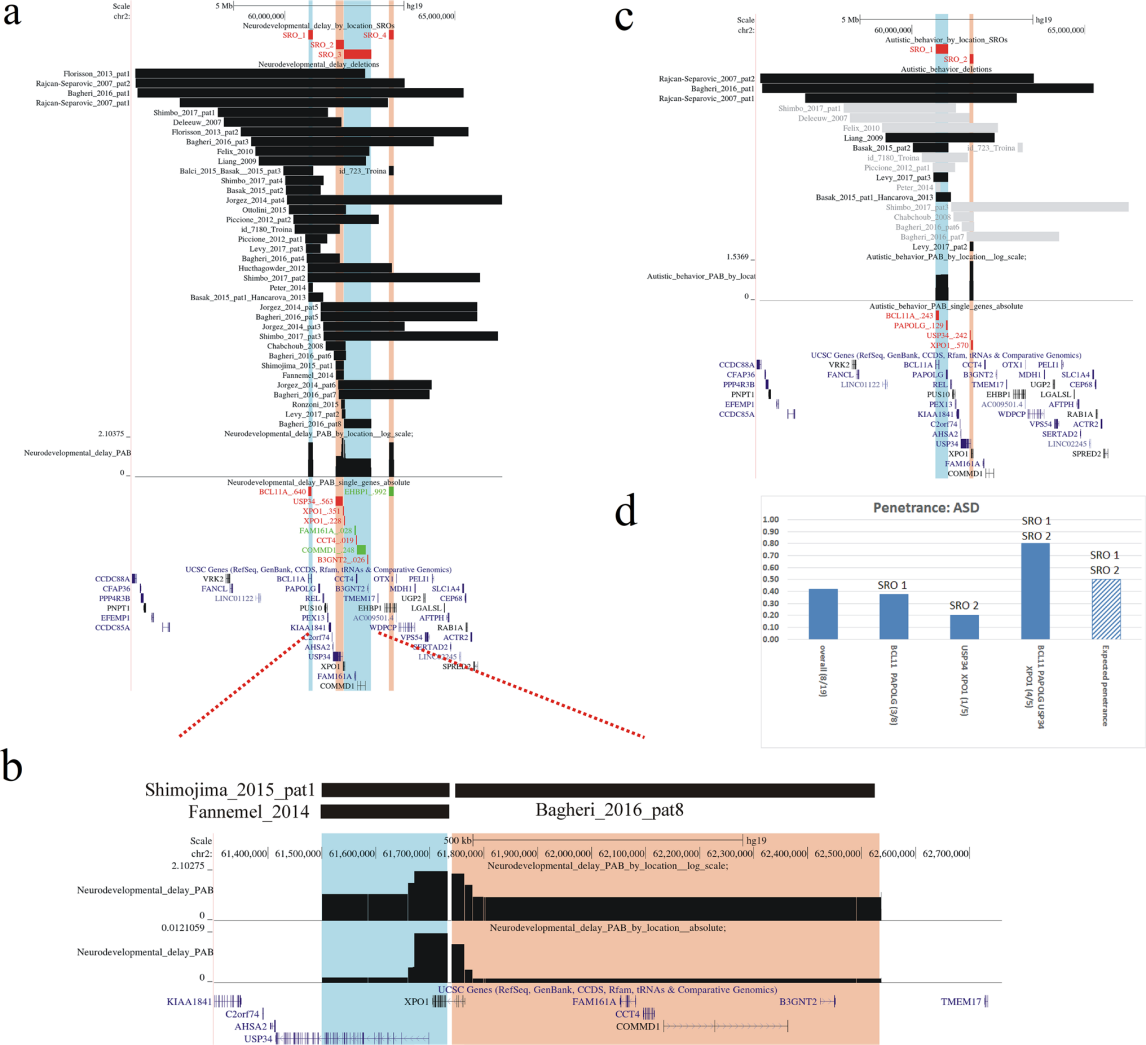
Visualization of SROs linked to Neurodevelopmental delay and autistic behavior. Regions included in the SROs are alternatively highlighted in blue and orange. The black and the gray bars indicate respectively deletions from patients with or without the selected clinical feature, among those assessed for the trait. The name of genes located into the SROs are written in red (pLI >  = 0.95) or in green (pLI < 0.95), along with the cumulative probability (cp) of the corresponding gene region to contain the disease locus. A graph displaying the estimated probability distribution in log scale of the genomic location of the disease loci inside each SRO is shown. **a** Neurodevelopmental disorders. All deletions are penetrant and outline three SROs containing 5 genes intolerant to loss − of − function variation (*BCL11A* in SRO1, *USP34* and *XPO1* in SRO2, *XPO1*, *CCT4* and *B3GNT2* in SRO3) while SRO4 only includes the *EHBP1* gene with a pLI score of 0.3. **b** Magnified view of the distribution of the probability of SRO2 and SRO3 in (A), both in log ratio and in absolute scale. In both SROs the probabilities corresponding to the genomic region of *XPO1* are approximately one order of magnitude greater than elsewhere inside SROs, indicating *XPO1* as the most likely contributor to the trait within SRO2 and SRO3. **c** Autistic behavior. Two SROs are depicted for this trait, SRO1 containing the *BCL11A* and *PAPOLG* genes, and SRO2 including the *USP34* and *XPO1* genes. **d** This trait shows an overall penetrance of 0.42, resulting from penetrance of deletions (blue boxes) involving solely SRO1 (0.38), or SRO2 (0.20), or both (0.8). The expected penetrance (box with blue diagonal lines) calculated according to the heterogeneity model of interaction (P_hm_) for deletions encompassing both SRO1 and SRO2 is 0.5, suggesting epistasis between genetic loci in SRO1 and SRO2

**Fig. 2:**
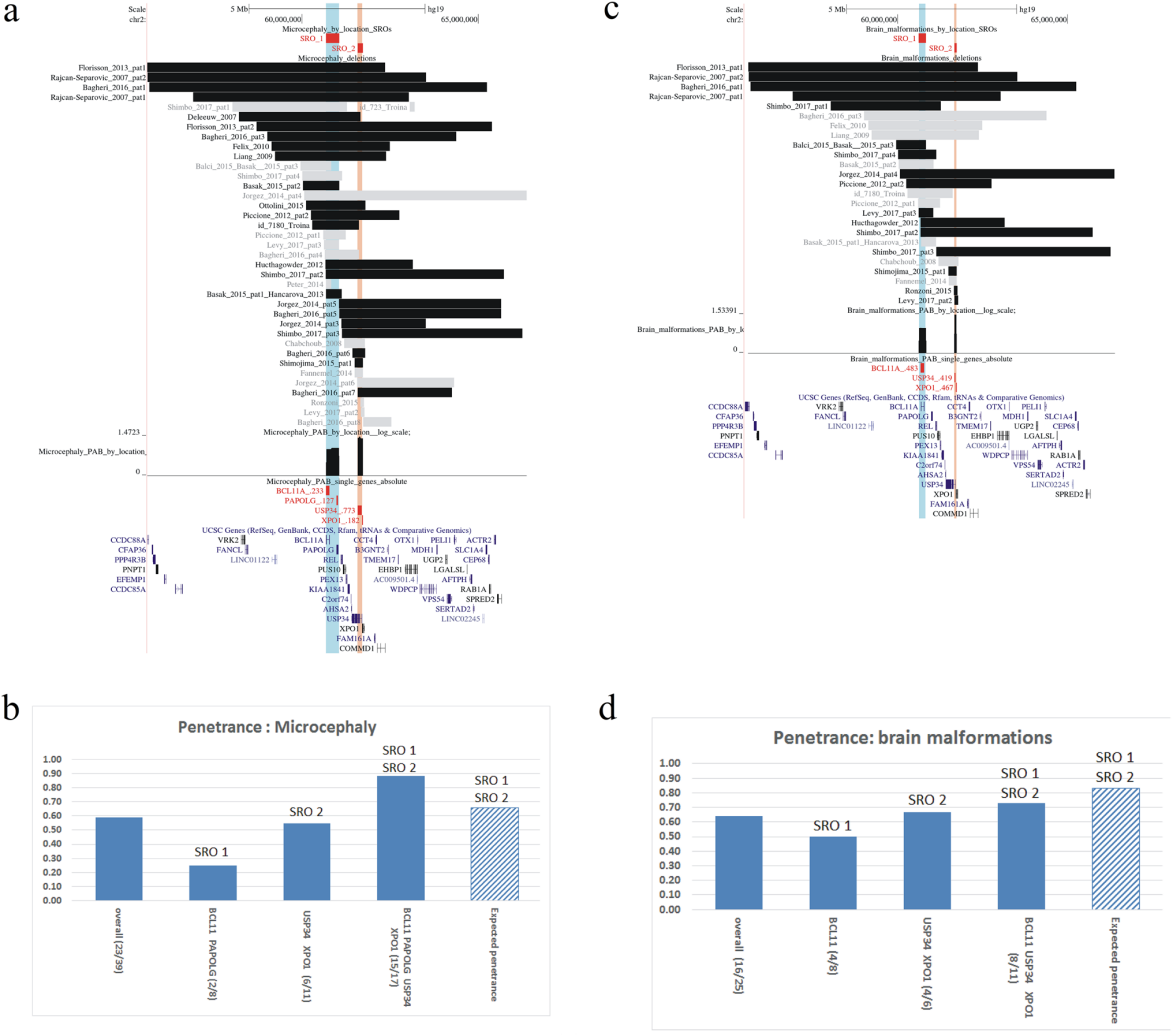
Visualization of SROs and penetrances associated with microcephaly (**a**, **b**) and brain malformations (**c**, **d**). **a**, **c** Regions included in the SROs are alternatively highlighted in blue and orange. The black and the gray bars indicate respectively deletions from patients with or without the selected clinical feature, among those assessed for the trait. The name of genes located into the SROs are written in red (pLI >  = 0.95) or in green (pLI < 0.95), along with the cumulative probability (cp) of the corresponding gene region to contain the disease locus. A graph displaying the estimated probability distribution in log scale of the genomic location of the disease loci inside each SRO is shown. **b**, **d** observed penetrances for deletions overlapping only a unique SRO or both SROs are represented by blue boxes, while the calculated penetrance p_hm_ is indicated by a dashed box. **b** The higher penetrance of SRO2 (0.55) in respect to SRO1 (0.25) may give evidence of a major role of *USP34* and *XPO1* genes in microcephaly whereas the observed penetrance of deletions encompassing both SROs (0.88) is higher than the expected p_hm_ (0.66), suggesting epistatic interaction between genes in both SROs. **d** Four penetrant deletions include exclusively SRO1 or SRO2, with different penetrances of 0.50 and 0.67 respectively. The expected p_hm_ (0.83) is slightly greater than the observed penetrance for deletions including both SROs (0.73), evoking an additive effect rather than epistasis for disease genes inside the SROs

**Fig. 3:**
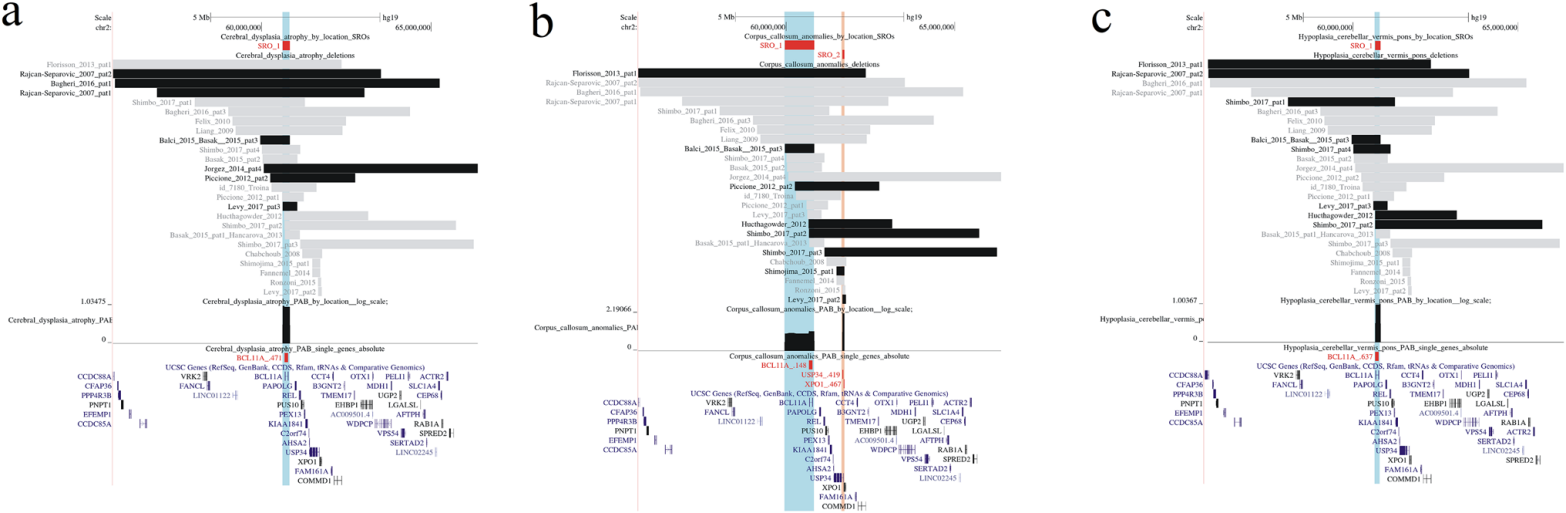
Visualization of the SROs linked to different brain anomalies **a** cortical dysplasia, **b** corpus callosum anomalies, and **c** cerebellar anomalies. Regions included in the SROs are alternatively highlighted in blue and orange. The black and the gray bars indicate respectively deletions from patients with or without the selected clinical feature, among those assessed for the trait. The name of genes located into the SROs are written in red (pLI >  = 0.95) or in green (pLI < 0.95), along with the cumulative probability (cp) of the corresponding gene region to contain the disease locus. A graph displaying the estimated probability distribution in log scale of the genomic location of the disease loci inside each SRO is shown. **a** A unique SRO containing the *BCL11A* gene is defined by 7 penetrant deletions resulting in a penetrance for cortical dysplasia of 0.37 (7/19). **b** Two SRO containing, respectively, the *BCL11A* and the *USP34* and *XPO1* genes are depicted for corpus callosum anomalies. SRO1 derives from a unique penetrant deletion of ~ 0.8 Mb and has a probability distribution profile showing a slight increase in the *BCL11A* corresponding region. The penetrance for deletions encompassing exclusively SRO1 is 0.12 (1/8) while that for deletions related to SRO2 is 0.4 (2/5). Four penetrant and seven non penetrant deletions overlap both SRO1 and SRO2, resulting in a penetrance for hemizygosity of both these regions of 0.36. **c** The unique SRO for cerebellar anomalies contains the *BCL11A* gene and arises from 8 penetrant deletions among a totality of 18 deletions overlapping this SRO, resulting in a penetrance of 0.44

**Fig. 4:**
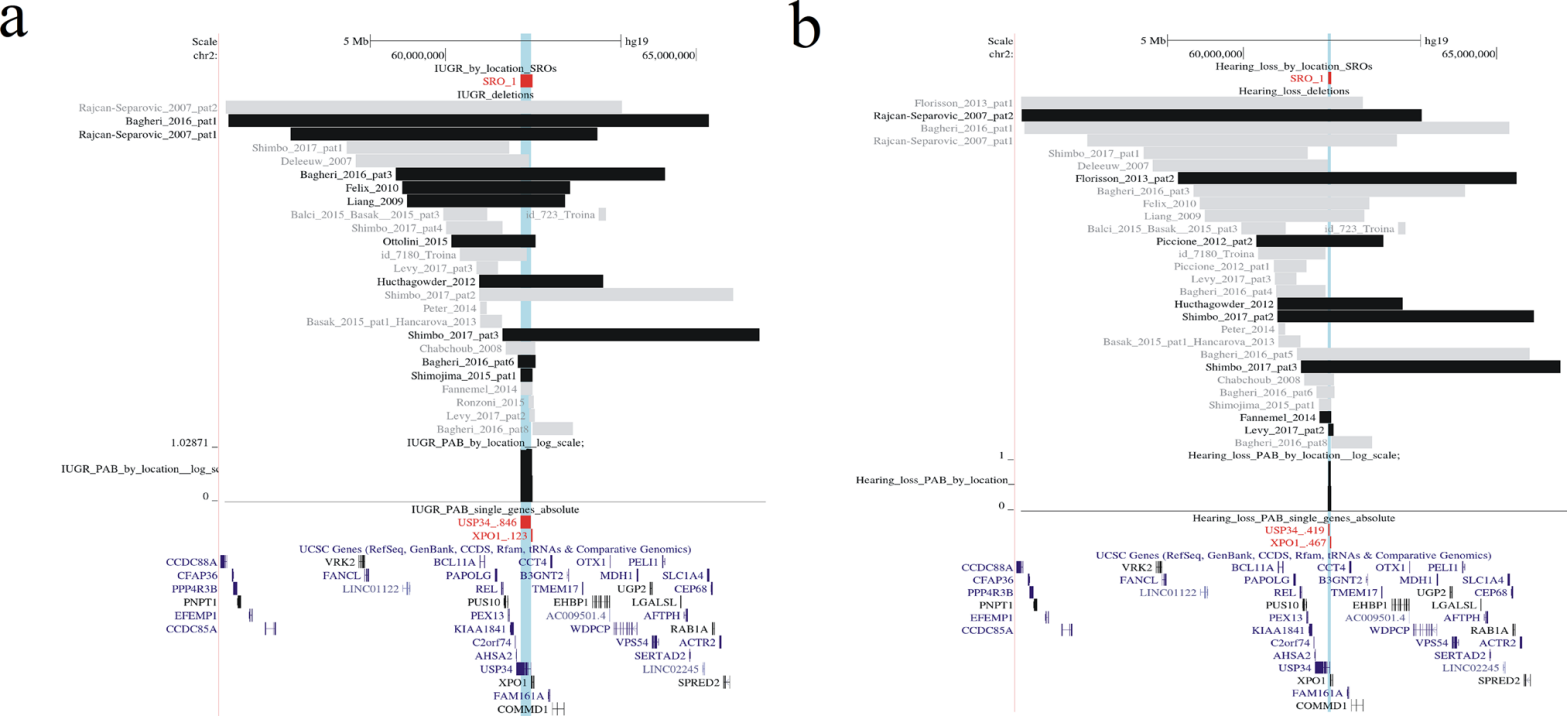
Visualization of SROs linked to intrauterine growth restriction (**a**), and hearing loss (**b**). Regions included in the SROs are highlighted in blue. The black and the gray bars indicate respectively deletions from patients with or without the selected clinical feature, among those assessed for the trait. The name of genes located into the SROs are written in red (pLI >  = 0.95) or in green (pLI < 0.95), along with the cumulative probability (cp) of the corresponding gene region to contain the disease locus. A graph displaying the estimated probability distribution in log scale of the genomic location of the disease loci inside each SRO is shown

A variable number (1–4) of SROs for each specific phenotypic feature were identified (see Figs. 1, 2, 3, 4). For almost all traits, except IUGR and hearing loss, our analysis pinpointed at least two SROs including the *BCL11A*, and the *USP34* and *XPO1* genes, respectively. Since these latter two genes are very close each other, they either mapped in a single sharp SRO or in two different larger SROs, depending on the set of overlapping penetrant deletions of the trait.

**Fig. 5:**
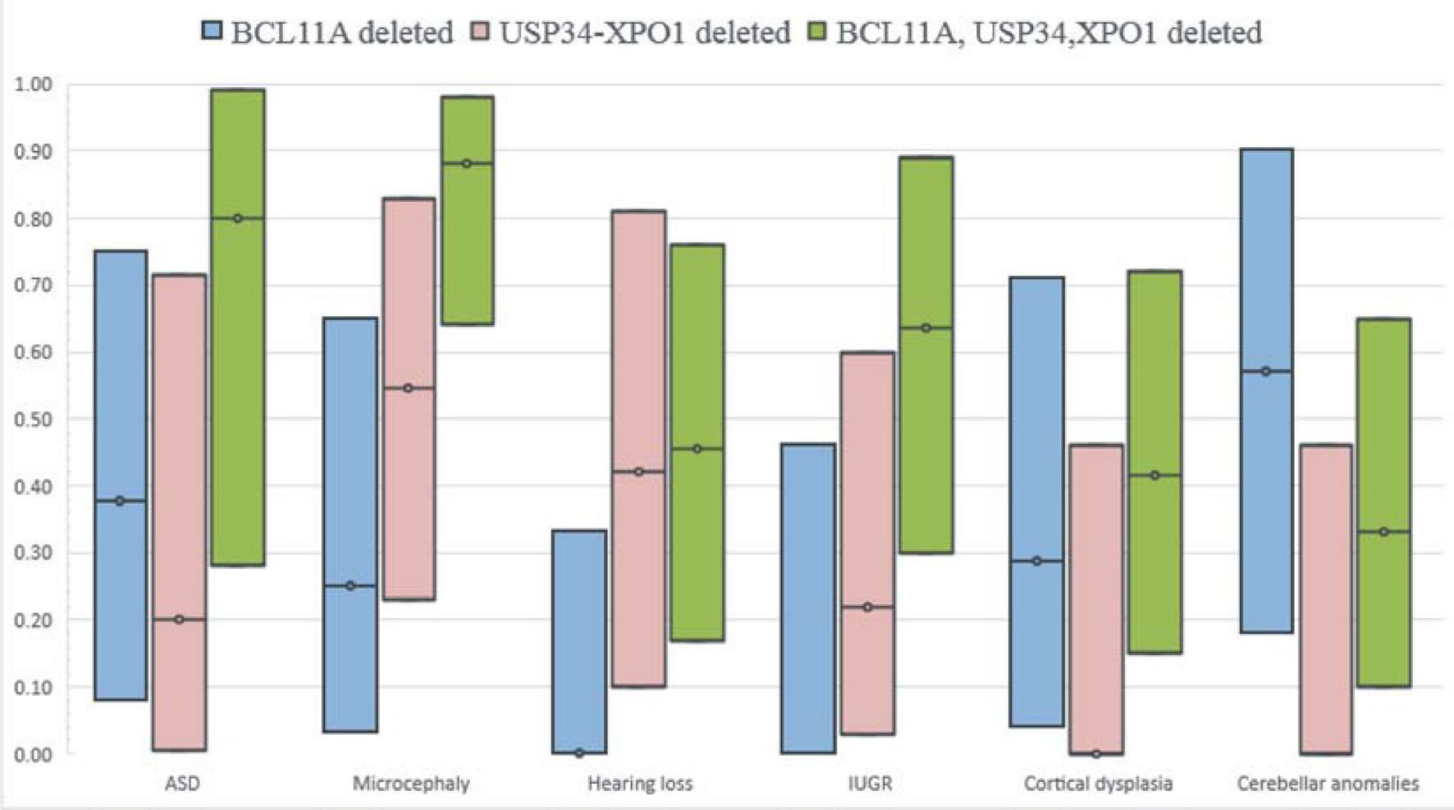
Penetrance of single or concurrent hemizygosity for the main driver genes in the 2p15p16.1 microdeletion syndrome. Black circled lines represent the estimated penetrance of deletions affecting the corresponding gene(s). Upper and lower boundaries of the colored boxes indicate the upper and lower limits of the 95% confidence intervals of the estimated penetrance

As anticipated by previous studies, this data confirmed that these three genes play a major role in most clinical features of the syndrome but also that many traits may be related to distinct genes. Considering each clinical feature separately (Figs. 1, 2, 3, 4, 5), it clearly emerges that deletions involving only a specific SRO often have different penetrances and that, besides showing epistasis or not, concurrent hemizygosity for genes inside different SROs led to the highest penetrance of the trait.

In the following sections, results for each clinical feature will be analyzed separately.

### Neurodevelopmental delay

This clinical feature was present in all patients whose deletions delineated 4 SROs (Fig. 1), three of which comprised 5 genes having pLI scores > 0.95, namely SRO1 (*BCL11A*), SRO2 (*USP34, XPO1*), SRO3 (*XPO1, CCT4, B3GNT2*) while SRO4 uniquely contained the *EHBP1* gene with a pLI score of 0.3. Of notice, *XPO1* region was splitted into two SROs (SRO2 and SRO3), due to the deletion of Bagheri et al. patient 8 having a distal margin inside the *XPO1* gene; however, we cannot exclude that a more precise definition of the breakpoint would have delineated a single narrow SRO involving *XPO1* only. While these two SROs include other haploinsufficiency intolerant genes, their probability distributions (Fig. 1 a, b) clearly demonstrated a remarkable increase in the regions corresponding to the *XPO1* gene suggesting that *XPO1* (SRO2 − SRO3) and *BCL11A* (SRO1) are the main responsible for that trait.

### Autistic behavior

Two SROs emerged for this trait (Fig. 1 c). The first one (SRO1; p: 3/8, 0.38) included the *BCL11A* (cp: 0.24) and the *PAPOLG* (cp: 0.18) genes while the second (SRO2; p: 1/5, 0.20) involved the *USP34* (cp: 0.24) and *XPO1* (cp: 0.579) genes. Interestingly, deletions involving both SROs showed a penetrance of 0.8 (4/5) greater than expected penetrance of 0.5 according to the heterogeneity model of interaction.

### Microcephaly

Penetrant deletions for microcephaly outlined two SROs (Fig. 2 a); SRO1 (p: 0.25, 2/8) including the *BCL11A* (cp: 0.23) and *PAPOLG* (cp: 0.13) genes, and SRO2 (p: 0.55, 6/11) overlapping the *USP34* (cp: 0.77) and *XPO1* (cp: 0.18) genes. Fifteen penetrant and two non − penetrant deletions completely or partially intersected both SROs, leading to an observed penetrance of 0.88 (15/17), while the expected penetrance p_hm_ was 0.66 (Fig. 2 b).

### Brain abnormalities

This clinical feature showed an overall penetrance of 0.64 (16/25) with penetrant deletions defining two SROs, respectively SRO1 (p: 0.5, 4/8) only including the *BCL11A* gene (cp:0.48), and SRO2 (p: 0.67, 4/6) containing the *USP34* (cp: 0.42) and *XPO1* (cp: 0.47) genes (Fig. 2 c). Rearrangements overlapping both SROs showed a penetrance of 0.73 (8/11) to be compared to a slightly greater p_hm_ of 0.83, suggesting an independent effect rather than epistasis of the two genomic regions (Fig. 2 d). To investigate whether single genes haploinsufficiency gave rise to different neuroradiological findings, we repeated the analysis after having introduced as additional characterizing traits cortical dysplasia, corpus callosum anomalies, and cerebellar hypoplasia. Our results are in accordance with the hypothesis reported by Shimbo et al. that *BCL11A* was related to cerebellar abnormalities, corroborating its pivotal role in neuronal development (Shimbo et al. 2017). The analysis clearly showed (Fig. 3) that *BCL11A* is involved with cortical and cerebellar anomalies whereas deletions of *USP34 − XPO1* genes are more likely to result in hypoplasia or agenesis of the corpus callosum.

### IUGR

A single SRO, comprising the *USP34* (cp: 0.84) and *XPO1* (cp: 0.12) genes, results from the overlap of 10 penetrant deletions associated with this clinical feature (Fig. 4 a). Deletions of this SRO display a penetrance of 0.55 (10/18) while the overall penetrance for this trait is 0.38 (10/26).

### Hearing loss

The trait has an overall penetrance of 0.28 (8/29). The unique SRO outlined for this feature involves the genes *USP34* (cp: 0.42) and *XPO1* (cp: 0.47) (Fig. 4 b) whose hemizygosity has a penetrance of 0.42 (8/19).

### Evaluation of the role of combined haploinsufficiency of the main driver genes on specific clinical features

Since our results clearly demonstrate that *BCL11A, USP34* and *XPO1* genes are involved in almost all clinical traits we studied, we decided to further analyze the penetrance of single or concurrent hemizygosity for these genes in clinical traits showing incomplete penetrance. While hampered by large and overlapping confidence intervals owing to the rarity of the microdeletion syndrome, the analysis showed that deletions simultaneously affecting all three genes are generally more penetrant than rearrangements involving only *BCL11A* or *USP34* and *XPO1* genes (Fig. 5). This is especially evident for the clinical features autistic behavior and microcephaly, further in agreement with a possible epistatic effect of the combined haploinsufficiency of these genes. Deletions affecting only the *BCL11A* gene are not penetrant for hearing loss and IUGR traits, while those uniquely involving the *USP34* and *XPO1* genes are neither penetrant for cortical dysplasia nor for cerebellar anomalies. However, in case of IUGR, deletions involving the three genes are remarkably more penetrant in respect of deletions which do not include the *BCL11A* gene, suggesting that this latter gene or other loci included in larger deletions may modulate this trait.

## Discussion

The most intuitive pathogenic mechanism in contiguous gene syndrome (CGS) concerns the involvement of dosage sensitive genes, leading to haploinsufficiency and consequently to an altered phenotype. Clearly, in a context of different sized deletions spanning a large genomic region, it often remains challenging to understand the precise role of specific loci among multiple genes (and genetic elements) in the onset of the observed phenotypes. A straightforward approach to the identification of candidate genes in CGS usually relies on focusing on known functional role, considering genes located in the genomic regions of minimal overlap between deletions in affected patients. Eventually, the identification of loss of function variants by sequencing studies and results coming from targeted functional assays, may further sustain the role of candidate genes. This simple approach is however challenged by several factors. Indeed, the delimitation of these genomic areas is less informative for traits showing incomplete penetrance both because fewer data are available for SRO mapping and because one cannot refine the critical region by excluding genomic segments corresponding to non − penetrant deletions interstitial to the SRO. Moreover, rather than haploinsufficiency of specific genes, the phenotype may either result from a long − range genomic dysregulation (Spielmann et al. 2018) or from complex interplays between hemizygous genes sharing biological pathways (Jensen and Girirajan 2019). At this regard, Andrews and colleagues have interestingly highlighted a higher functional similarity between genes in pathogenic CNVs in respect to genes in benign CNVs, further supporting that complex pathogenic mechanisms may underlie the clinical outcome in CGS (Andrews et al. 2015). In a similar scenario, reduced support from sequencing studies should be expected, as the more complex the pathogenic mechanism, the less likely becomes the identification of inactivating mutations in candidate genes for cardinal features of CGS. Moreover, functional analysis that suppress single candidate genes in animal model may not recapitulate the human phenotype. To complicate this picture, the phenotype may be further modulated by the individual genetic background (Pizzo et al. 2018).

The 2p15p16.1 microdeletion syndrome is a genetic disorder characterized by several different clinical features, most of them displaying incomplete penetrance and that frequently occur in patients carrying non overlapping deletions, indicating that different genomic segments are individually involved in the same clinical trait. To improve the genotype/phenotype correlation in this complex syndrome, we have investigated the cohort of 2p15p16.1 patients by parallel, trait − driven analyses to delineate genomic regions associated with six selected clinical features using a bayesian probabilistic method, which also takes into account information derived from non − penetrant deletions (Fichera et al. 2020).

**Fig. 6:**
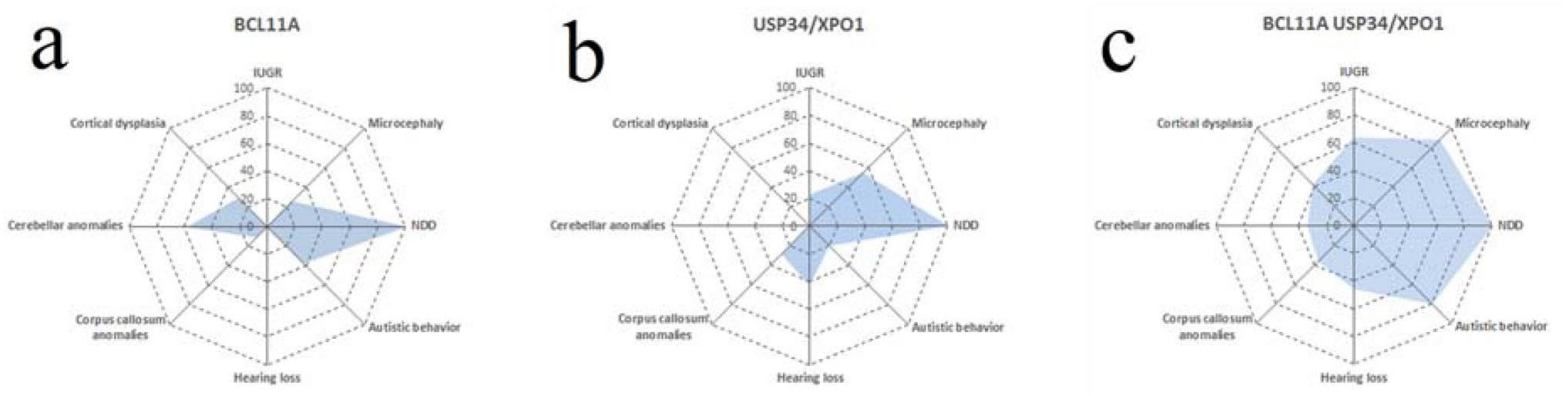
Radar plot showing SROs penetrances for different clinical signs. The penetrances are calculated for deletions encompassing the SROs including the genes **a** *BCL11A* alone, **b** *USP34/XPO1* alone, and **c** both *BCL11A* and *USP34/XPO1*

In agreement with previous studies, our analysis confirmed that the main driver genes for most clinical traits of the syndrome are *BCL11A*, and *USP34/XPO1,* the latter two genes being too close to each other to be included in different SROs. However, our probabilistic analysis greatly favors *XPO1* over *USP34* as candidate gene, at least for neurodevelopmental delay (Fig. 1B)*.* This finding is in keeping with functional assay on zebrafish model where the knockdown of the ortholog of *USP34* did not show any obvious developmental defects (Bagheri et al. 2016). In agreement with previous reports (Shimbo et al. 2017), our investigation showed that *BCL11A* is particularly involved in cortical and cerebellar anomalies, and autistic behavior whereas *USP34/XPO1* deletions are more associated with corpus callosum anomalies, microcephaly, IUGR, and hearing loss (Fig. 6). Furthermore, our analysis also suggests that the final clinical outcome is largely modulated by a complex interaction between candidate genes.

*BCL11A* is a subunit of the mammalian BAF SWI/SNF ATP − dependent chromatin remodeling complex (Kadoch et al. 2013; Simon et al. 2020) and a transcriptional repressor of fetal hemoglobin (Sankaran et al. 2008). Causative variants in genes coding for BAF − associated subunits have been found in subjects with different forms of neurodevelopmental disorders (Ciernia et al. 2017; Bögershausen and Wollnik 2018). Wiegreffe and colleagues demonstrated that *bcl11a* controls cell polarity and radial migration of upper layer cortical neurons and that deletion of *bcl11a* in mice results in hypoplasia of superficial neocortex (Wiegreffe et al. 2015).

Dias et al. recently showed that loss of function mutations of *BCL11A* cause a clinical syndrome (Dias − Logan syndrome) characterized by ID, dysmorphic features and persistence of fetal hemoglobin. Other less frequent phenotypic traits of the syndrome are microcephaly, autistic behavior, and cerebellar and corpus callosum anomalies (Dias et al. 2016). These findings further support the direct role of the hemizygosity of *BCL11A* in the 2p15p16.1 deletion syndrome.

*USP34* encodes for a deubiquitinase that acts as a positive regulator of Wnt signaling by promoting nuclear stabilization and accumulation of *AXIN1* and *AXIN2* (Lui et al. 2011); it also plays a role in genome stability by promoting ubiquitin signaling at DNA double − strand breaks (Sy et al. 2013), and is required for osteogenesis and bone formation (Guo et al. 2018) and inhibits osteoclastogenesis by regulating NF − κB signaling (Li et al. 2020).

*XPO1 (*also known as *CRM1)* is a member of the nuclear export family of proteins and mediates the transport of large macromolecules across the nuclear membrane to the cytoplasm (Fornerod et al. 1997). *XPO1* has been linked to the occurrence of axonal damage (Kim et al. 2010; Li et al. 2013) and has been found upregulated in multiple sclerosis (Haines et al. 2015). It has also been shown that suppression of *Xpo1* induces apoptosis of the cortical neural progenitors in mouse (Li et al. 2020).

On the contrary to *BCL11A*, albeit thousands of whole − exome or whole − genome sequencing studies on patients with neurodevelopmental disorders, no inactivating mutations in *USP34* or in *XPO1* genes have yet been reported in the literature. While the rarity of deleterious alleles may account for these findings, other hypotheses should also to be considered. (1) Since among deletions which do not involve *BCL11A* only one (Bagheri patient 8) affects exclusively *XPO1* and not *USP34*, one cannot exclude that concurrent deletion of both genes may be generally required to trigger the disease. (2) The pathogenic mechanism may be linked to the disruption of a regulatory element in the genomic regions of *USP34* and *XPO1* genes, rather than to haploinsufficiency for one or both of them. The first hypothesis greatly depends on a possible functional convergence of *USP34* and *XPO1* that may justify a possible complex effect of haploinsufficiency of these genes. Interestingly, it has been shown (Bagheri et al. 2016) that pairs of orthologs of human *USP34/XPO1* and *BCL11A/REL* colocalize in zebrafish, suggesting a possible functional relationship between some genes within the 2p15p16.1 region. While this may support the fact that deletions disrupting both *USP34* and *XPO1* could have a higher penetrance than deletions or small inactivating mutations on one of them, making less likely to find single gene defects in sequencing analyses, further data are needed to clarify this point.

Many clinical traits characteristic of the syndrome have incomplete penetrance. We showed that deletions both involving *BCL11A* and *USP34/XPO1* are generally more penetrant than those affecting only *BCL11A* or *USP34/XPO1* genes (Fig. 5 and Fig. 6). Clearly, deletions that concurrently involve genes associated on their own with a specific trait are more likely to produce the phenotypic feature whatever the nature of their interaction. Nevertheless, the comparison between the observed penetrance and that predicted when considering concurrent haploinsufficiencies acting independently on a specific trait, may reflect a more complex model than the additive one. Although more cases would have been needed to obtain better estimates of penetrances (Fig. 5), our investigation supports an epistatic effect between candidate genes, especially for traits such as autistic behavior and microcephaly where the observed penetrance of deletions encompassing both SROs is remarkably greater than that expected for genes acting independently (Fig. 1 d and Fig. 2 d).

Functional convergence on shared biological pathways is a prerequisite for genes to interact. Interestingly, *BCL11A* and *USP34/XPO1* are all related to the Wnt signaling pathway which plays a crucial role throughout all stages of brain development (Noelanders and Vleminckx 2017; Bem et al. 2019). *BCL11A*, involved in dendritic cells differentiation, is downregulated by non − canonical Wnt signaling pathway that inhibits dendritic cells differentiation (Xiao et al. 2016). *BCL11A* orthologous in mice has as a downstream target frizzled related protein 3, a modulator of Wnt signaling, whose dysregulation impacts on dorsal spinal neurons development (Yin et al. 2019). *XPO1* is involved in the nucleus − cytoplasmic shuttling of *APC*, regulating *β − catenin* availability (Turner and Sullivan 2008; Li et al. 2010). *USP34* through its deubiquitinase activity stabilizes *AXIN1* and *AXIN2*, regulating positively *β − catenin* transcriptional activity (Lui et al. 2011).

We should also point out that for larger deletions encompassing different SROs, hemizygosity for other genes or regulatory elements between or close to SROs may potentially contribute to selected phenotypic features. At this regard, it has been demonstrated in zebrafish embryos that the suppression of the expression of the ortholog of *REL*, a gene mapping between both main SROs, results in microcephaly (Bagheri et al. 2016). While functionally validated in zebrafish, the role of *REL* in the 2p15p16.1 syndrome remains unclear because biallelic mutations of *REL* inherited from healthy parents have recently been identified in patients suffering from a severe form of immunodeficiency, however with no cognitive disability nor brain anomalies (Beaussant − Cohen et al. 2019; Lévy et al. 2021). Two de novo missense variants in the *CCT4* gene, coding for a molecular chaperone known to play a role in the folding of actin and tubulin, were identified in ASD probands from the Simons Simplex Collection (Iossifov et al. 2014). Interestingly, this gene is included in the four penetrant deletions encompassing SRO1 and SRO2 (Fig. 1 c) and could be considered as a potential risk factor for autistic behavior in the syndrome.

The SRO containing the gene *EHBP1* (EH domain − binding protein 1) is defined uniquely by the small deletion in our patient 2 (Id_723). *EHBP1* encodes for a protein known to be involved in endocytic trafficking. It colocalizes with the actin cytoskeleton and its overexpression leads to actin reorganization (Guilherme et al. 2004). This protein is implicated in prostate cancer and in early development, especially in Drosophila, where it plays an essential role in eye development (Rai et al. 2020). Considering that *EHBP1* is not predicted to be intolerant to loss − of − function mutation (pLI = 0.35) and that no other deletions encompass exclusively this gene, we cannot exclude that the deletion is coincidental to the phenotype of the patient. Clearly, the role of this gene, if any, should be confirmed by additional cases.

In conclusion, the 2p15p16.1 microdeletion syndrome, as probably the majority of the CGS, should be regarded as complex genetic disorder where the fully penetrant clinical traits are driven by few major genes while the constellation of other, less penetrant, phenotypic signs are due to a complex cooperation between genetic loci with functional convergence. Variable size of deletions interesting different set of genes, together with variants in the individual genetic background, probably dictates the final clinical outcomes. In this context, dissecting the penetrance for single clinical traits, may improve the genotype/phenotype correlation and may help to identify specific pathogenic mechanisms in CGSs.

## Data Availability

Data supporting our findings and the software are available on request.
